# CARE 1000: randomized controlled trial for the evaluation of the effectiveness of a mHealth app for supporting the first 1000 days of life

**DOI:** 10.1186/s13063-022-06953-y

**Published:** 2022-12-12

**Authors:** Laura Brunelli, Sofia Bussolaro, Margherita Dal Cin, Luca Ronfani, Sara Zanchiello, Andrea Cassone, Giuseppa Verardi, Raffaella Dobrina, Michele Bava, Tamara Stampalija

**Affiliations:** 1 Department of Medical, Surgical and Health Sciences, University of Trieste, Trieste, Italy; 2 Department of Prevention, Giuliano Isontina Healthcare University Trust, Trieste, Italy; 3 Clinical Epidemiology and Healthcare Services Research, Institute for Maternal and Child Health - IRCCS “Burlo Garofolo”, Trieste, Italy; 4 Area Science Park, Trieste, Italy; 5 Healthcare Professions Department, Institute for Maternal and Child Health - IRCCS “Burlo Garofolo”, Trieste, Italy; 6 Unit of Fetal Medicine and Prenatal Diagnosis, Institute for Maternal and Child Health - IRCCS “Burlo Garofolo”, Trieste, Italy; 7 ICT Services, University of Trieste, Trieste, Italy

**Keywords:** mHealth, First 1000 days, Health prevention

## Abstract

**Background:**

Recent developments in eHealth and mobile health (mHealth), as well as the introduction of information and communication technology innovations in clinical practice, such as telemedicine, telemonitoring, and remote examinations, are already changing the current scenario and will continue to generate innovations in the coming decades. The widespread use of mobile devices, with an estimated nearly 30 billion devices and more than 325,000 apps worldwide, will provide various opportunities for people to take control of their own health. Already in 2017, most of the apps available were focused on pregnancy support, more than any other medical field. There have been some reported experiences with social media and mHealth that could benefit the promotion of maternal physical and mental health during pregnancy. However, many apps targeting the first 1000 days of a child’s life do not consider the continuity between the prenatal and postnatal periods and their joint impact on maternal and child health. The aim of this study is to evaluate the effectiveness of this mHealth app to support women during the first 1000 days (from conception to 24 months of age) and to improve health prevention behaviours such as immunizations during pregnancy, weight gain during pregnancy, abstinence from smoking and alcohol consumption, and adherence to the routine childhood immunization schedule. In addition, the study aims to understand the level of appreciation of this mHealth app as a tool to overcome information and communication gaps between patients and institutions.

**Methods:**

Conduction of a randomized controlled trial.

**Discussion:**

Our results will be relevant for improving this mHealth app to promote health and prevention and to support the first 1000 days of life for both mother and child. Our results will be relevant to the future expansion of such an mHealth app to promote positive health-related outcomes in patients and co-user satisfaction and to support the organization of health services.

**Trial registration:**

ClinicalTrials.gov NCT05500339.

**Supplementary Information:**

The online version contains supplementary material available at 10.1186/s13063-022-06953-y.

## Administrative information

Note: the numbers in curly brackets in this protocol refer to SPIRIT checklist item numbers. The order of the items has been modified to group similar items (see http://www.equator-network.org/reporting-guidelines/spirit-2013-statement-defining-standard-protocol-items-for-clinical-trials/).

**Table:** 

Title {1}	CARE 1000. Randomized controlled trial for the evaluation of the effectiveness of a mHealth App for supporting the first 1000 days of life
Trial registration {2a and 2b}	Trial identifier: NCT05500339; registry name: ClinicalTrials.gov
Protocol version {3}	18/03/2022; version n.2
Funding {4}	ARGO system, a protocol agreement signed in 2018 among the Friuli Venezia Giulia Region, the Italian Ministry of Education University and Research, and the Italian Ministry of Economic Development funded the app development. This work was supported by the Ministry of Health, Rome—Italy, in collaboration with the Institute for Maternal and Child Health - "IRCCS Burlo Garofolo", Trieste – Italy.
Author details {5a}	LB, SB[Department of Medical, Surgical and Health Sciences, University of Trieste]; MDC [Department of Prevention, Giuliano Isontina Healthcare University Trust]; LR [Clinical Epidemiology and Healthcare Services Research, Institute for Maternal and Child Health - IRCCS "Burlo Garofolo" ]; SZ [Area Science Park]; AC. GV, RD [Healthcare Professions Department, Institute for Maternal and Child Health - IRCCS "Burlo Garofolo" ]; TS [Department of Medical, Surgical and Health Sciences, University of Trieste; Unit of Fetal Medicine and Prenatal Diagnosis, Institute for Maternal and Child Health - IRCCS "Burlo Garofolo" ], MB [ICT Services, University of Trieste].
Name and contact information for the trial sponsor {5b}	This work is supported by the Ministry of Health, Rome—Italy, in collaboration with the Institute for Maternal and Child Health IRCCS Burlo Garofolo, Trieste – Italy.
Role of sponsor {5c}	This study is supported by the Institute for Maternal and Child Health - "IRCCS Burlo Garofolo", Trieste, Italy, through the contribution given by the Italian Ministry of Health (Ricerca Corrente 04/22). Patients will be enrolled at the Institute for Maternal and Child Health - "IRCCS Burlo Garofolo". The role of the sponsor is to ensure that proper arrangements are in place to initiate and manage the study and to hold indemnity insurance and legal liability. Furthermore, the project that lies behind the study is funded by Area Science Park within the complex project “ARGO System,” a protocol agreement signed in 2018 among the Friuli Venezia Giulia Region, the Italian Ministry of Education University and Research, and the Italian Ministry of Economic Development.

## Introduction

### Background and rationale {6a}

Recent developments in eHealth and mobile health (mHealth), as well as the introduction of information and communication technology innovations in clinical practice, such as telemedicine, telemonitoring, and remote examinations, are already changing the current scenario and will bring about further innovations in the coming decades [1]. The widespread use of mobile devices, with an estimated nearly 30 billion devices [2] and more than 325,000 apps worldwide [3], will provide various opportunities for people to take control of their own health [4–6]. Already in 2017, most of the available apps focused on pregnancy support, more than any other medical field [7]. These mobile technologies for pregnancy support have also expanded the opportunities for parents and parents-to-be to self-manage health issues [8]. Results from a recent study conducted in Switzerland in 2019 showed that 91% of parents reported using digital media to search for information about their child’s health and development [8]. Some social media and mHealth experiences have been shown to be beneficial in promoting maternal physical and mental health during pregnancy [4]. However, many apps targeting the first 1000 days of life do not consider the continuity between the prenatal and postnatal periods and their joint impact on maternal and child health [9]. Moreover, the reliability, quality, and effectiveness of available pregnancy and postnatal care apps are still unclear. The potential negative consequences of misleading healthcare and lifestyle information or unnecessary worry and stress during pregnancy are of concern because women are more sensitive to external influences [10, 11]. Given the wide variability of apps in terms of ownership, responsibility, and updating of content, as well as the degree of reliability of the source [12], and the lack of a standardized certification system [12, 13], this limited evidence for their effectiveness is not surprising [10]. Furthermore, the standard design and development of apps do not usually take into account the level of health literacy of the target audience [13, 14], leaving the choice of which mHealth app to use to the preferences of users in terms of aesthetic, functional, and engagement aspects, regardless of the validity of the content provided or privacy and security considerations [15]. Considering that the first 1000 days of life represent a critical window of time for the child development, and given the critical influence of the health information source on maternal well-being, lifestyle, and decision-making related to pregnancy and child health [16], we have classified this as a burning issue that warrants further investigation.

### Objectives {7}

People are increasingly using eHealth and mobile health solutions thanks to the ease of access and rapid communication digital solutions provide. While this brings a variety of benefits and new opportunities, it may also pose a threat to public health in terms of prevention and health promotion by exposing them to potential risks such as lack of control and fake news.

The authors seek to answer the following research questions:

- Is an mHealth app a potential tool to bridge information and communication gaps between patients and institutions in the first 1000 days of life?
- What are the health and social implications of a patient- and family-centred mHealth application in a maternal and child health institute?
- Could such a tool be useful for supporting pregnant women and new mothers in the first 1000 days of life?
- Could such an mHealth tool be used at a regional or national level to improve the relationship between citizens and healthcare providers?

The primary aim is to evaluate the effectiveness of the mHealth app in supporting women in the first 1000 days (from conception to 24 months of life) and improving health behaviours, e.g. immunizations during pregnancy, weight gain during pregnancy, abstaining from smoking and alcohol consumption, and adherence to the routine childhood immunization schedule.

The secondary aim is to explore the level of appreciation of this mHealth app as a tool to bridge information and communication gaps between patients and healthcare providers.

Our findings will be relevant to improving this mHealth app to promote health and prevention and support the first 1000 days of life for mothers and children. Our findings will be relevant to the future expansion of such an mHealth app to promote positive health-related outcomes for patients and co-user satisfaction and to support the organization of healthcare services.

### Trial design {8}

Randomized, open-label, controlled trial to prospectively evaluate the superiority of care delivered with the support of the mHealth app over standard care.

## Methods: participants, interventions, and outcomes

### Study setting {9}

The study population is pregnant women attending the Unit of Fetal Medicine and Prenatal Diagnosis for first-trimester echography during pregnancy. Enrolment and subsequent data collection will be performed at Unit of Fetal Medicine and Prenatal Diagnosis, Institute for Maternal and Child Health - IRCCS "Burlo Garofolo", Trieste, Italy; analyses will be performed by Clinical Epidemiology and Healthcare Services Research, Institute for Maternal and Child Health - IRCCS "Burlo Garofolo", Trieste, Italy.

### Eligibility criteria {10}

Inclusion criteria are as follows: age ≥ 18 years, good understanding of the Italian language, possession of a smartphone for the app download, and willingness to deliver at the Institute for Maternal and Child Health - IRCCS "Burlo Garofolo", Trieste, Italy.

Exclusion criteria are as follows: individuals with cognitive impairments and individuals unable to give consent in person.

### Who will take informed consent? {26a}

Participants will be asked by the researcher to provide informed consent for study participation and data collection and management in accordance with the European General Data Protection Regulation (GDPR) and Italian law D.Lgs. 101/2018.

### Additional consent provisions for collection and use of participant data and biological specimens {26b}

In the consent form, participants are asked for permission for the research team to use and share data in aggregate form or from supervisory authorities, as relevant. No biological samples will be collected as part of this study.

### Explanation for the choice of comparators {6b}

Standard care was chosen as the comparator because pregnant women in clinical practice are usually educated by health professionals about preventive health and health promotion (e.g. to improve their preventive behaviours such as immunizations during pregnancy, weight gain during pregnancy, abstaining from smoking and alcohol consumption, and adherence to the routine childhood immunization schedule).

### Intervention description {11a}

Both groups of women (A and B) will be asked to complete an initial questionnaire to collect demographic and other data at time zero (T0). This paper questionnaire includes questions on health literacy, knowledge, and attitudes toward prevention and health promotion behaviours. At the same time, women in the experimental group will be assisted to download and instal the mHealth app on their smartphone or tablet.

During the study period, women in group A will be able to use the app, which was developed in collaboration with Area Science Park to support the first 1000 days of life, with all its features and content, while women in group B will be exposed to standard care. Specific content will be presented to the user according to the current pregnancy trimester or postpartum period following a scheduled routine; in any case, all content will always be available for free consultation throughout the usage period. Links to relevant institutional websites for further information will also be provided. The app will also include a FAQ (frequently asked question) section and a calendar function with the ability to set appointments and reminders. The app’s content and topics will include information on health preventive behaviours such as immunizations during pregnancy, weight gain during pregnancy, smoking abstinence and alcohol use, and adherence to routine childhood immunization schedules. The app will be an informative and educational tool aimed at improving health behaviours and well-being.

Three additional questionnaires will be collected from both groups of women during the study period at the end of the second and third trimesters (T1–2) and during the postpartum period (T3). While the second and third questionnaires will be distributed in printed form according to the women’s planned access to the Unit of Fetal Medicine and Prenatal Diagnosis, IRCCS "Burlo Garofolo", the fourth questionnaire will be made available online on the IRCCS "Burlo Garofolo" platform and in the app itself. Women will be reminded to complete it through the app’s calendar function and through email and phone reminders.

At the end of the trial period, women will also be asked to rate their appreciation of the app features and content by providing feedbacks in digital form and participating in focus group sessions.

Data on app use (frequency of app access, content consultation, references consultation, use of app features, etc.), access to and use of health services, and vaccination adherence during pregnancy will also be collected with support from the regional health information systems.

### Criteria for discontinuing or modifying allocated interventions {11b}

If any of the following clinical situations occur during the study period in women already enrolled, this will be grounds for exclusion of the patient from the study: intrauterine death, severe foetal malformation, miscarriage, and therapeutic abortion.

### Strategies to improve adherence to interventions {11c}

Women will be reminded to complete questionnaires via the app’s calendar function and by email and phone.

### Relevant concomitant care permitted or prohibited during the trial {11d}

Participation in the study does not require any change in usual care pathways (including taking medications or providing standard health promotion and prevention information), and these will be maintained for both study arms.

### Provisions for post-trial care {30}

There is no anticipated harm and compensation for study participation.

### Outcomes {12}

The primary study outcome is the differences between experimental and control groups regarding adherence to DPT vaccination in pregnant women (questionnaires; evaluation at T3—postpartum period). The choice of this outcome is due to the clear evidence of the need for maternal vaccination, especially against pertussis, which can be a life-threatening infection for the newborn. Moreover, available data on DPT vaccination coverage among pregnant women in Trieste for the past years show an unsatisfactory percentage of women vaccinated with DPT (only 55%).

Secondary study outcomes are the differences between experimental and control groups in:

- Knowledge and behaviours in terms of health prevention and promotion, especially regarding folic acid intake, weight gain during pregnancy, smoking and alcohol consumption, breastfeeding, adherence to the routine childhood immunization schedule (questionnaires; evaluation at T1—end of the second trimester, T2—end of the third trimester, T3)
- Level of health literacy (Italian version of the 16-item European Health Literacy Survey Questionnaire HLS-EU-Q16; evaluation at T1, T2, and T3)
- Evaluation of the social and health impact of the introduction of an mHealth app for a maternal and child hospital (questionnaire; evaluation at T3)

As with the experimental group, the final evaluation will include:

- The appreciation of such a tool to bridge the information gap between patients and health institutions (qualitative satisfaction questionnaire, freely modified from the Mobile Application Rating Scale (MARS); evaluation at T3)
- Analysis of data on app use collected by the app itself throughout the study period

### Participant timeline {13}

Pregnant women will participate in the study from the first antenatal echography until the end of the first 1000 days of their child’s life. The evaluation time points are enrolment in the study (T0), end of the second trimester (T1), end of the third trimester (T2), and the postpartum period (T3). Data on app use will be collected throughout the study period. Other variables collected (T0) include data describing the population and potential confounders (e.g. age, country of origin, marital status and composition, socioeconomic level, educational level) and risk/protective factors (e.g. smoking and alcohol habits before pregnancy, data on current pregnancy).

Data collection will be based on self-completed questionnaires in paper form (T0, T1, T2) and online (T3), as well as data collected using information and management systems, and data on app use.

The questionnaires are provided as a Supplementary Material to this study protocol. Additional data to confirm vaccination adherence and to monitor access to and outcomes of preventive health, health promotion, and care can be accessed through the regional health information systems.

### Sample size {14}

Given that 55% of pregnant women living in Trieste received the DPT vaccine during pregnancy in 2018, and assuming that this rate increases to 70%, a sample size of 163 women per group (total of 326 women) with an alpha of 0.05 and a beta of 0.2 is required. Considering the possibility of spontaneous abortion and the possibility of losing participants during the follow-up period, the investigators determined to recruit 180 women per group (360 women in total).

### Recruitment {15}

Women attending the Unit of Fetal Medicine and Prenatal Diagnosis, IRCCS "Burlo Garofolo", in Trieste for the first echography of their pregnancy (around 12° gestational week (gw)) will be screened according to the inclusion and exclusion criteria outlined previously. The women concerned will be offered participation in this study. Recruitment will be kept open until the required sample size is reached; recruitment is expected to last 3.5 months. Since all women living in the area of Trieste will be required to visit the IRCCS "Burlo Garofolo" public health institute in Trieste for the first pregnancy echography, this will allow the researchers to reach the required number of participants.

## Assignment of interventions: allocation

### Sequence generation {16a}

Randomization will be open and will consider enrolment days. Eligible participants will be randomly assigned in a 1:1 ratio to the intervention group (use of the mHealth app) or to the control group (standard care). Enrolled women will be randomly assigned to two groups (the experimental group (A) and the control group (B)) using PC-driven randomization of enrolment days. This will facilitate enrolment as the researcher can give more information and train the groups of women to download and initialize the app.

### Concealment mechanism {16b}

Casualty of randomization will be ensured by hiding the randomized sequence in opaque and appropriately numbered envelopes.

### Implementation {16c}

The researcher creates the random order, and participants are then enrolled according to the randomized tag.

## Assignment of interventions: blinding

### Who will be blinded {17a}

None, this is an open-label study.

### Procedure for unblinding if needed {17b}

Unblinding is not required because this is an open-label study.

## Data collection and management

### Plans for assessment and collection of outcomes {18a}

Baseline data on all women invited to participate will be collected in a datasheet to control for unwanted bias in language, age, or other variables at enrolment. Data collection will be based on self-completed questionnaires in paper form (T0, T1, T2) and online (T3), together with data collected through information and management systems and data about app use. An English-translated version of the questionnaires is provided a Supplementary material to this study protocol. Additional data to confirm vaccination adherence and to monitor access to and outcomes of preventive health, health promotion, and care can be accessed through the regional health information systems.

### Plans to promote participant retention and complete follow-up {18b}

Women will be reminded to complete questionnaires via the app’s calendar function and via email and phone reminders.

### Data management {19}

Data entry for the printed questionnaires will be possible through a digital interface similar to that used for data collection during follow-up via the online survey. Each participant is linked to a unique participant code; duplicates will be checked after data entry. Data will be then merged with other sources, the utilization data, and the health information system to obtain the final complete data set.

### Confidentiality {27}

All participants are assigned a specific ID code that will be used throughout the study to track all relevant information and questionnaires to ensure pseudo-anonymization.

### Plans for collection, laboratory evaluation, and storage of biological specimens for genetic or molecular analysis in this trial/future use {33}

See the section ‘Additional consent provisions for collection and use of participant data and biological specimens {26b}’, no biological samples will be collected.

## Statistical methods

### Statistical methods for primary and secondary outcomes {20a}

Categorical variables will be presented as absolute numbers and percentages; continuous variables will be presented as mean and standard deviation or median and interquartile range, depending on data distribution assessed visually and using the Kolmogorov–Smirnov test.

The main population characteristics of the participating women in both groups (age, marital status, living area, country of origin, socioeconomic level, educational and occupational level, etc.) will be compared to assess the effectiveness of randomization. Differences between the two groups on categorical outcomes will be assessed using the chi-square test or Fisher’s exact test, as appropriate. Comparisons between continuous variables will be made with nonparametric Student *T* or Mann–Whitney tests, as appropriate for the data distribution. The main analysis will be an ‘intention-to-treat design’: all randomized women with an available main outcome will be assigned to the associated group regardless of intervention. A per-protocol analysis will also be performed. A statistically significant difference will be considered to exist if the *p*-value is < 0.05.

### Interim analyses {21b}

We do not anticipate problems during study conduct, as there are no risks associated with app use that require formal study discontinuation. Therefore, an interim analysis is not planned.

### Methods for additional analyses (e.g. subgroup analyses) {20b}

No additional analysis is planned.

### Methods in analysis to handle protocol non-adherence and any statistical methods to handle missing data {20c}

The main analysis will be performed as an ‘intention-to-treat’. All randomized women with an available main outcome will be analysed in the group to which they were randomized, regardless of whether they received the assigned intervention. Reasons for dropping out of the study will be detailed, and the potential impact of missing data on study outcomes will be explored through sensitivity analysis.

### Plans to give access to the full protocol, participant-level data, and statistical code {31c}

The data set and statistical code analysed as part of the current study will be available from the corresponding author upon reasonable request, as is the full protocol.

## Oversight and monitoring

### Composition of the coordinating centre and trial steering committee {5d}

IRCCS "Burlo Garofolo" of Trieste will be responsible for the day-by-day support of the study through the Unit of Fetal Medicine and Prenatal Diagnosis.

### Composition of the data monitoring committee, its role, and reporting structure {21a}

The DMC is composed of a researcher from Area Science Park and a researcher from IRCSS "Burlo Garofolo" who have no conflict of interest.

### Adverse event reporting and harms {22}

Available data suggest that serious adverse events are not expected. Any adverse events, even if not related to the use of the app, but occurring during study conduct, will be reported to the relevant regulatory authorities in accordance with national legislation, specifying seriousness, severity, and causality.

### Frequency and plans for auditing trial conduct {23}

Regular meetings (monthly/bimonthly) will be held to monitor the study conduct and address potential problems.

### Plans for communicating important protocol amendments to relevant parties (e.g. trial participants, ethical committees) {25}

Any changes to the study protocol will be communicated to the IRCSS "Burlo Garofolo" Institutional Review Board and the FVG Regional Ethical Committee responsible for approving the study.

### Dissemination plans {31a}

The results of the study will be disseminated to the scientific community, relevant stakeholders, and the public through publications, public communications, and social media posts.

## Discussion

Despite the unpredictability of the COVID-19 pandemic in the next few years, support from this mHealth tool will be a constant; furthermore, access to maternal and child health care will not be reduced because these services are considered essentials. Loss during the follow-up period is likely the greatest risk for this study. Several reminder measures have already been planned to address this risk, including email, phone calls, and reminders via the app’s calendar.

## Trial status

Not yet recruiting. Time to recruit since start: 3.5 months. Recruitment will end before March 31, 2023.

## Supplementary Information

**Additional file 1. **CARE 1000 questionnaires.

## Data Availability

All data will be accessible to researchers at IRCSS "Burlo Garofolo", the University of Trieste, and Area Science Park. Relevant data will be available to the scientific community and the public through publication. Any data required to support the protocol can be made available upon request.
