# Supplementary material for: CARE 1000: randomized controlled trial for the evaluation of the effectiveness of a mHealth app for supporting the first 1000 days of life
Source: Trials. 2022 Dec 12;23:1007. doi: 10.1186/s13063-022-06953-y (PMC9744031; doi:10.1186/s13063-022-06953-y)
Supplement: Supplementary file 1 — Additional file 1. CARE 1000 questionnaires. [file 13063_2022_6953_MOESM1_ESM.docx]

**CARE1000 questionnaires**

**(T0 only) General information**

- In what year were you born? ___________
- What week of pregnancy are you currently in? ___________week + ___________days
- What is the zip code of the place where you live? ___________
- In which country were you born? Italy / Other (please specify)_______________
- Your mother tongue is: Italian / Other (please specify)_______________
- To which of the following ethnic groups do you feel you belong? Italian / Greek / Iberian / North and West European / North African / Balkan / Asian / Other (please specify)_____________
- What is the highest level of schooling you have achieved? None (not yet) / Elementary school / Junior High School / High school / University / Postgraduate (Master's/Doctorate) / Other (please specify)__
- How many years did you attend school? ________________ years
- What is your current occupation? Executive Businesswoman / Freelancer / Employee / Worker / Housewife / Student / Not employed or unemployed / Other (please specify)___________
- Your occupation is: Full-time / Part-time or part-time
- Are you currently: Single / Married or cohabiting / Divorced / Widowed
- Your current economic situation is: Excellent / Good / Sufficient / Insufficient / I do not want to answer
- What was your weight before your pregnancy? _____kg
- What is your height? ________m
- Did you smoke during your pregnancy? Yes/No
- Did you smoke in the months before you became pregnant? Yes / No
- Have you had alcoholic beverages in the past? (Yes even if it was only half a glass of wine/small beer/alcoholic aperitif) Yes / No
- Have you been vaccinated against papillomavirus (HPV)? Yes / No
- Is your current partner(s) the biological father of the child? Yes / No / I prefer not to answer
- Your current partner is: Male / Female / I prefer not to answer
- In what year was your partner born? _______________
- Where was your partner born? Italy / Other (please specify)_______________
- Your partner's native language is: Italian / Other (please specify)_______________
- To which of the following ethnic groups does your partner feel he/she belongs? Italian / Greek / Iberian / North and West European / North African / Balkan / Asian / Other (please specify)_____
- What is the highest level of schooling your partner has completed? None (not yet) / Elementary school / Junior High School / High School / University / Post-university (Master's/Doctorate) / Other (please specify)________________
- How many years has your partner/partner attended school? ________________ years
- What is your partner’s current occupation? Executive Entrepreneur / Freelancer / Employee / Worker / Homemaker / Student / Not employed or unemployed / Other (please specify)________
- What is your partner's occupation: Full-time / Part-time or part-time
- Does your partner smoke? Yes / No
- This pregnancy was: Planned / Spontaneous / Occurred after medically assisted reproductive techniques / Other (please specify)_________________________
- This pregnancy is: Singleton / Twin (2 twins) / Twin (3 or more twins)
- During this pregnancy, you plan to be cared for: By your midwife as part of the physiological pregnancy pathway / By your private gynaecologist / Other (please specify)__________
- Do you already have other children? No / 1 / 2 / 3
- Have you ever heard of folic acid? Yes / No
- Did you take folic acid before pregnancy? Yes / No

**(T0, T1, T2, T3) Monitoring habits and behaviours**

- Has your work situation changed since the onset of pregnancy? No, situation has not changed / I have lost my job / I am currently on maternity leave / Other (please specify)__________
- (T3 only) What is your current occupation? Businesswoman / Freelancer / Employee / Worker / Housewife / Student / Not employed or unemployed / Other (please specify)__________
- (T3 only) Your occupation is: full-time / part-time
- (T3 only) Your current economic situation is: Excellent / Good / Sufficient / Unsatisfactory / Would not like to answer
- What is your current weight? ________kg
- (T3 only) Are you taking any medications? Please specify_________________
- Do you currently smoke? Yes/No
- How many cigarettes do you smoke per day? __________no
- Are you exposed to second-hand smoke? Yes / No
- (T3 only) Does your partner currently smoke? Yes / No
- (T3 only) Did you smoke during pregnancy? No / Yes / 2-3 times/week / 4 or more times/week
- How often have you had alcoholic beverages during your pregnancy so far? (Consider even half a glass of wine/small beer/alcoholic aperitif) Never / 1-2 times/month / 3-4 times/month / 2-3 times/week / 4 or more times/week
- During your pregnancy so far, how often did you drink 4 or more units of alcoholic beverages at one time? (By alcohol unit, we mean one glass of wine or one can of beer or one aperitif or one shot of liquor) Never / 1-2 times/month / 3-4 times/month / 2-3 times/week / 4 or more times/week
- The following are considered alcoholic units: a glass of wine, a can of beer, a shot of liquor). According to this definition, you are currently: a teetotaller / Stopped drinking alcohol when you found out you were pregnant / Take 1-2 alcohol units per week / Take 1 alcohol unit per day / Take 1-3 alcohol units per day / Take more than 3 alcohol units per day
- Have you taken folic acid in the last few months? Yes / No
- (T3 only) Have you taken any other supplements in the last few months? Yes / No
- Do you plan to have your son/daughter vaccinated in the future? Yes, for all vaccinations / Yes, but only for some vaccinations / Have not decided / Have not thought about it yet / No
- (T1, T2, T3 only) Have you been vaccinated against pertussis during this pregnancy (DPT vaccine: diphtheria, tetanus, pertussis)? Yes/No
- (T3 only) Are you currently breastfeeding? Yes, exclusively / Yes, but with supplemental feeding of breast / No / Other (please specify)_________

**(T0, T1, T2, T3) Current knowledge and opinions**

Select the applicable answer (true/false) for each of the statements:

- Maternal breastfeeding
  - May prevent obesity in infants
  - Should be discontinued if infant colic occurs
  - Promotes “mother's return to form"
  - prevents early respiratory tract illnesses in the baby/child
- Alcohol consumption during pregnancy
  - can lead to changes in the development of the foetus
  - can have harmful effects already at conception
  - if you limit yourself to moderate amounts of alcohol (e.g., half a glass per meal), there is no risk of harm
- Smoking during pregnancy
  - may influence the occurrence of severe malformations in the child
  - may influence the premature birth of the child
  - may affect low birth weight of the child
  - may affect the occurrence of some early respiratory diseases of the child
- The intake of folic acid
  - may reduce the risk of premature birth
  - may reduce the baby’s risk of developing certain heart defects
  - may reduce the baby’s risk of developing certain neural tube defects such as spina bifida
- In your opinion, what is the recommended maximum weight gain during pregnancy for a normal-weight woman? ___kg
- How long is it recommended to exclusively breastfeed your baby (i.e., without adding solid foods or liquids)? There is no specific time / No. months______ / Do not know
- Until what age do you think it is desirable for your child to be breastfed? Until the time slid foods are introduced / Up to 1 year / Up to 2 years or even beyond

On a scale of 1 to 5, please indicate how much you agree with the following statements, where 1=not at all agree and 5=very much agree

- The disease being vaccinated against is less dangerous than the vaccine itself
- Too many vaccines are given together
- It is important to vaccinate children because the diseases that are vaccinated against can have very serious consequences
- If people stop vaccinating, many diseases that are now very rare could come back into circulation
- Healthy lifestyles can prevent disease without vaccinating children

**(T0, T1, T2, T3) Health literacy assessment.** Italian version of the 16-item European Health Literacy Survey Questionnaire HLS-EU-Q16

**(T3 only) Satisfaction questionnaire**

- Do you think the information conveyed by the app is complete? Not at all / Slightly / Somewhat / Very / Completely
- Do you think the information conveyed by the app is useful? Not at all / Slightly / Somewhat / Very / Completely
- Do you think the information conveyed by the app is easy to understand? Not at all / Slightly / Somewhat / Very / Completely
- Which of the topics offered by the app did you find most useful? __________
- Among those proposed by the app, are any of the topics covered in an unclear way? Yes/No
- If yes, which ones? __________
- Are there any topics not offered by the app that you would have liked to have explored? Yes/No
- If yes, which ones? __________
- How often have you searched the web (search engines, social media, social networks, blogs, sites, etc.) for information because you were not satisfied with the information offered by the app? Never / Rarely / Sometimes / Often / Always
- Have you experienced any issues while using the app? Yes/No
- If yes, which ones? __________

Express your degree of agreement/disagreement with each of the following six statements on a scale of 1 to 5, where 1 = Strongly disagree to 5 = Strongly agree.

- This app has increased my awareness of the importance of adopting behaviours for my health and the health of my child(ren) during pregnancy and puerperium.
- This app has increased my knowledge/understanding of behaviours for my health and that of my child(ren) to take during pregnancy and puerperium.
- The app has changed my attitude by improving behaviours for my health and that of my child(ren) during pregnancy and puerperium.
- The app increased my intentions/motivation to engage in behaviours for my health and that of my child(ren) during pregnancy and puerperium.
- This app encouraged me to seek further help to undertake behaviours for my own health and that of my child(ren) during pregnancy and puerperium (if there was a need).
- Using this app has increased my behaviours for my health and that of my child(ren) during pregnancy and puerperium.
- Overall, how satisfied are you with the app? Not at all / Slightly / Somewhat / Very / Completely
